# The Impact of SARS-CoV-2 Outbreak on the Polish Dental Community’s Standards of Care—A Six-Month Retrospective Survey-Based Study

**DOI:** 10.3390/ijerph18031281

**Published:** 2021-01-31

**Authors:** Bartosz Dalewski, Lukasz Palka, Paweł Kiczmer, Ewa Sobolewska

**Affiliations:** 1 Chair and Department of Dental Prosthetics, Pomeranian Medical University, 70-111 Szczecin, Poland; bartosz.dalewski@pum.edu.pl (B.D.); rpsobolewski@wp.pl (E.S.); 2 Private Dental Practice, 68-200 Żary, Poland; 3 Department and Chair of Pathomorphology, Faculty of Medical Sciences in Zabrze, Medical University of Silesia, 40-752 Katowice, Poland; pawel.kiczmer@protonmail.com

**Keywords:** COVID-19, SARS-CoV-2, epidemiology, delivery of health care, dental care, community dentistry, Poland, infection transmission, dentist–patient

## Abstract

Currently, SARS-CoV-2 is the primary pathogen worldwide, disrupting most of our everyday activities. The study aim was to evaluate its impact on the Polish dental community, standards of care, health, and welfare. Methods: A Google Forms survey was conducted among 303 dental practitioners. Results: Of respondents, 54.93% curbed the number of patients in the last six months, 34.21% declared no changes, and 10.86% reported an increase; whereas 70.7% of the respondents reported a treatment price increase within the same period (27.96% and 1.32% reported no changes and a decrease, respectively). Of the respondents, 15.5% did not close their businesses during the first wave of the pandemic. Most declared 1 or 2 month break, 30.7% and 34.7%, respectively. Some reported 3, 4, or 5 month breaks (15.84%, 1.32%, and 0.99%, respectively), and only two respondents (0.66%) did not admit patients at all. Headache episodes were more frequent among female dentists before the pandemic; after the pandemic, headache frequency increased among both sexes. Temporomandibular disorders (TMDs) were more frequent among women (*p* = 0.017). Conclusions: Most Polish dentists followed SARS-CoV-2 recommendations and restricted their practices to admitting only patients with pain or incomplete treatment. Decreased sleep parameters, head, back, and neck pain, were observed. This situation may affect dental health conditions in Polish society over time.

## 1. Introduction

The SARS-CoV-2 virus started to spread drastically around the world in December 2019. Reportedly, it took around two months to reach Europe and another month to reach Poland, resulting in the pandemic’s outbreak there. Because of its high infectivity, rapid transmission, mortality rates, and unpredictable treatment outcomes, most governments were underprepared to face its direct impacts, as well as possible long-term effects [1]. Currently, controlling the SARS-CoV-2 infections is the primary action worldwide, disrupting most of our everyday activities as it threatens humanity [2]. This positive-stranded (+) RNA virus spreads from an infection source to a healthy individual via respiratory droplets as a result of sneezing or coughing [3]. The virions pass through the host’s upper respiratory tract, then travel into the lung cells by exploiting angiotensin-converting enzyme 2 (ACE2) and transmembrane serine protease 2 (TMPRSS2). Urogenital transmission and its impact is widely discussed, mostly due to possible water contamination [4]. One of the most dangerous SARS-CoV-2 infection features is asymptomatic contagious carriers spreading the virus to healthy persons in their vicinity, e.g., workplaces, schools, shopping areas, or public transport. As the world prepares for vaccine deployment, new viral strains with mutations in spike proteins were found at the end of 2020 [5].

In Poland, no work sectors were left unaffected by the pandemic, including healthcare. As major hospital departments were instructed and accessorized with safety protocols, decontamination procedures, and personal protective equipment (PPE), the private sector, especially dental practices, were neglected and left entirely alone to deal with the problem [6]. No explicit government indications and safety protocols for dental care were initially issued [7]. The Polish Dental Association information campaign failed miserably [8], as many different reactions have been observed in the dental society, varying from locking down dental practices to ignoring the virus threat. In Poland, from 3January to 4:13p.m. CET, 22December 2020, there were 1,214,525 confirmed cases of COVID-19 with 25,783 deaths [9]. Currently, 42,425 people are licensed to provide dental services in Poland, out of whom almost 12,500 are active dentists [10]. Among this group, 9248 dentists are 65+ years of age and, therefore, at risk of a severe course of SARS-CoV-2 infection (Supplement 1). According to the Ministry of Health, 1610 dentists have been infected with SARS-CoV-2 so far, and eight have died [11]. Practitioners who decided to work at the beginning of the pandemic had to organize their practices thoroughly according to unclear safety protocols and independently equip themselves with hardly available PPEs. Due to the shortage, PPE prices skyrocketed quickly, up to 300% and more in comparison to pre-pandemic valuation, which led to additional cost increases and put even more strain on the dental business [6,9]. For the duration of the epidemic, the NHF (National Health Fund) proposed to increase the valuation of healthcare procedures by an average of less than 10% only in relation to the lowest valued emergency care services, which eventually settled with 3% more in terms of dental treatment. From the beginning of the virus outbreak, many dental organizations and discussion groups tried to determine, define, and clarify treatment principles on how to safely provide proper dental care for patients in need under such extreme conditions [8]. The only state recommendations available at that time were brief and concise. Issued by the Polish Dental Association, it stood as: “due to high risk of virus transmission associated with dental aerosol produced by handpieces and ultrasound scalers, procedures should be limited to those cases which are strictly necessary for pain reduction or to complete started treatments only” [11]. Because of the uncertainty of workplaces, the shortage of PPE availability, and the lack of clear regulations on working during the pandemic, this stressful situation could impact the dentists’ health and life quality [12,13].

The main goals of this survey-based study were to evaluate the impact of the pandemic outbreak on the Polish dental community’s standards of care, personal health, and welfare.

## 2. Materials and Methods

### 2.1. Study Design and Population

The survey was conducted between November and December 2020 among Polish dental practitioners. The tool used for data collection was a specifically designed online GoogleForms questionnaire. A representative sample group of dentists was gathered through Facebook community groups dedicated exclusively for Polish dentists: Dentyści Przypadki, Kursy i Dyskusje; Dentyści; Dentyści Ogłaszają; Stomatologia bez tajemnic.

### 2.2. Questionnaire

The questionnaire consisted of 30 questions with single or multiple choices, as shown in Supplement 2. It was anonymous with the first three questions concerning sex, age, and years of service. In questions 4 to 15, we asked about dental practices and working environments in reference to the pandemic period. The last fifteen questions concerned health conditions and life quality in two time periods, i.e., before and after 20 March 2020 (Supplement 2).

### 2.3. Data Gathering and Statistical Analysis

The results were collected by the research group and analyzed by a calibrated examiner who was unaware of the identity of the participants. Microsoft Excel (Microsoft 2019, Redmond, WA, USA) sheets were used to create tables and graphs. Three members of the research group independently reviewed the extracted data for accuracy. The chi-square test was used to explore the association between the parameters, and a *p*-value < 0.05 was considered statistically significant. The calculation was performed using Statistical Package for the Social Sciences, software version 22.0 (IBM Corp, Armonk, NY, U.S.).

### 2.4. Study Group

The study group consisted of 207 women (68.32%) and 96 men (31.68%). The mean age was 39.31 ± 10.43 years. The mean length of practice was 14.31 ± 10.29 years.

### 2.5. Statistical Analysis

The qualitative data are presented as the number of cases and percent. The chi-square test was performed to evaluate the association between qualitative variables. The quantitative data are presented as mean ± SD. ANOVA for repeatable measurements was performed to compare quantitative variables, considering the influence of sex. Due to a semi-quantitative scale describing temporomandibular disorder (TMD), Kendall’s tau coefficient was used to determine its association with quantitative variables describing stress levels, pain levels, and sleep quality. Statistical analysis was performed using RStudio software (Integrated Development for R. RStudio, PBC, Boston, MA, USA) [14].

## 3. Results

**Figure 1:**
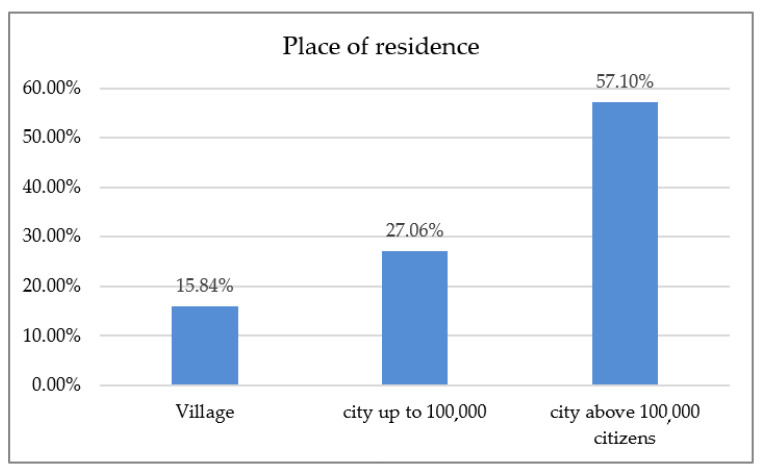
Place of residence.

The respondents’ place of residence is presented in Figure 1. Twenty-four of the respondents declared a village as their only place of practice (7.92%) and 279 dentists worked in the city. The most frequent profile of performed procedures was conservative dentistry and endodontics (Table 1).

**Figure 2:**
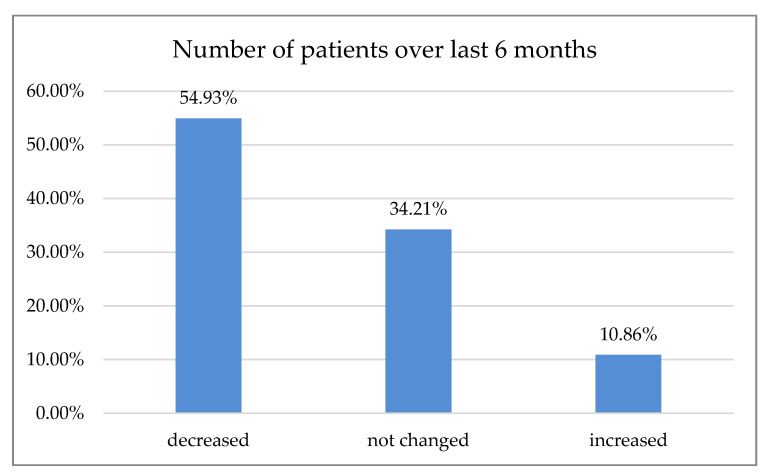
Changes in the number of patients during the last six months.

**Figure 3:**
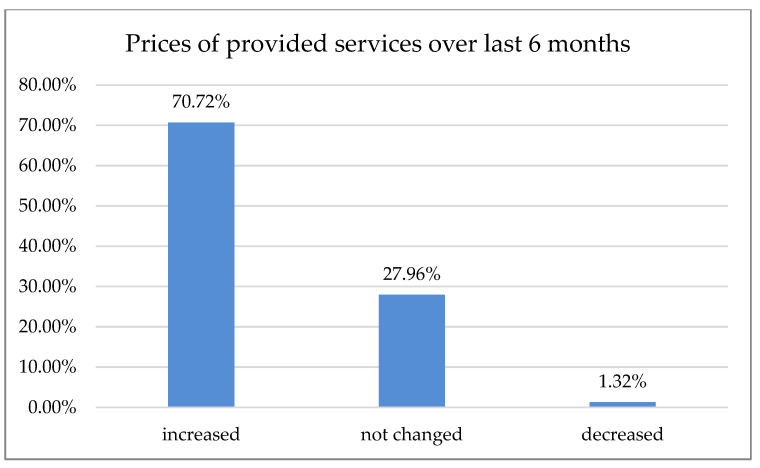
Changes in prices during the last six months.

Over 50% of the respondents declared that the number of patients decreased over the last six months (Figure 2); 70.72% of respondents reported an increase in prices over the last six months (Figure 3).

**Figure 4:**
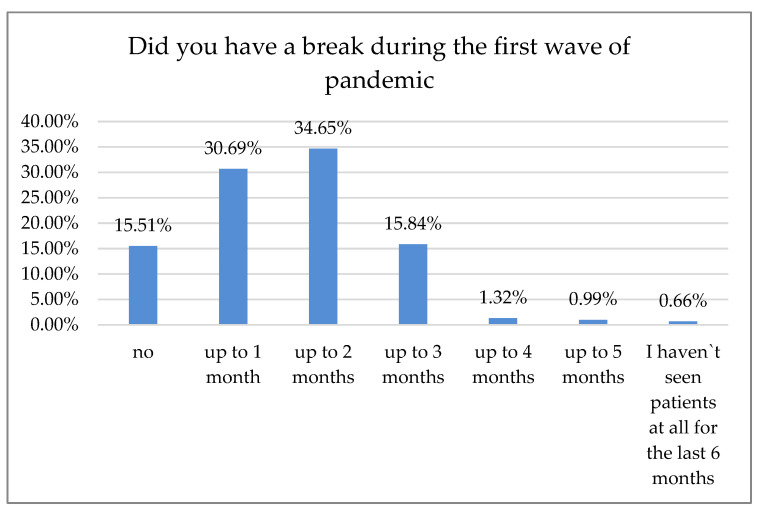
The length of a break from work declared by respondents.

Forty-seven dentists declared that they had no break during the first wave of the pandemic. Most of the respondents reported 1 or 2 month break during the first wave of the pandemic (Figure 4).

**Figure 5:**
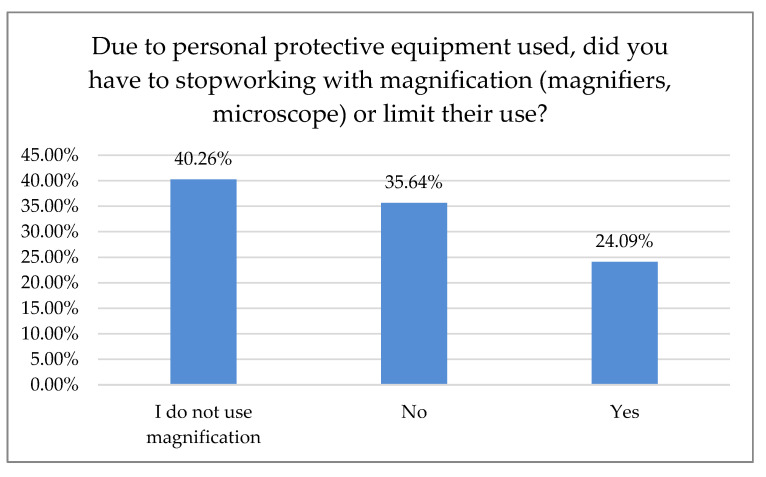
Usage of magnification during the pandemic.

Of the respondents, 24.09% reported that they had to cease working with magnification due to personal protection (Figure 5).

**Figure 6:**
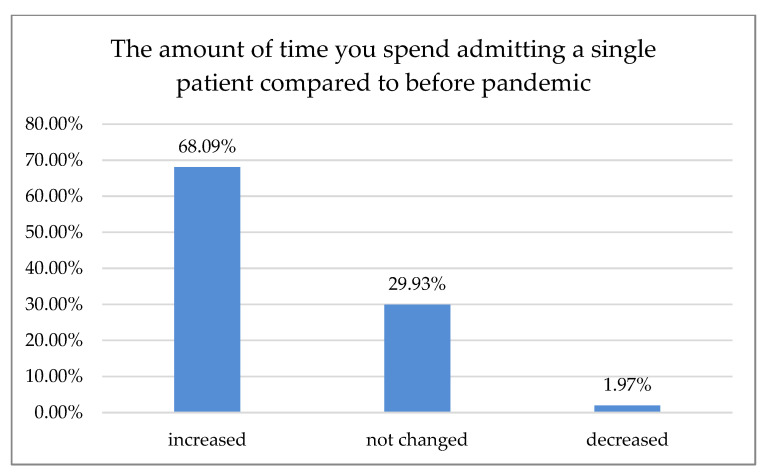
Amount of time spent per one patient during the pandemic.

Of the respondents, 68.09% reported that the amount of time spent admitting a single patient increased (Figure 6).

**Figure 7:**
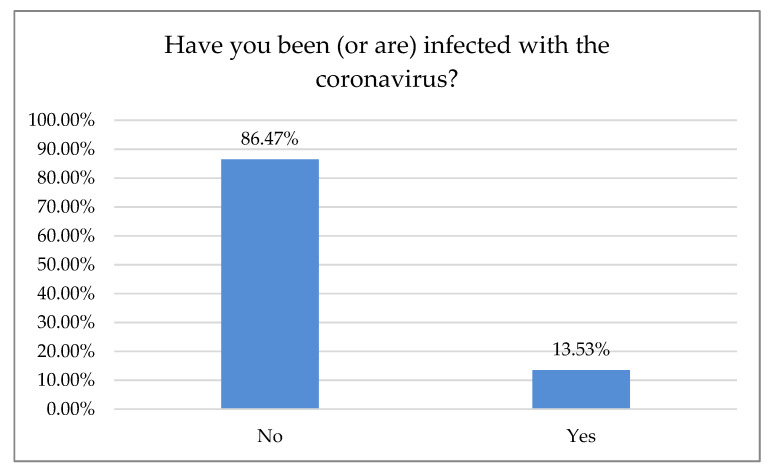
The number of COVID-19 cases among examined dentists.

Of the interviewed dentists, 13.53% were infected with the coronavirus (Figure 7).

**Figure 8:**
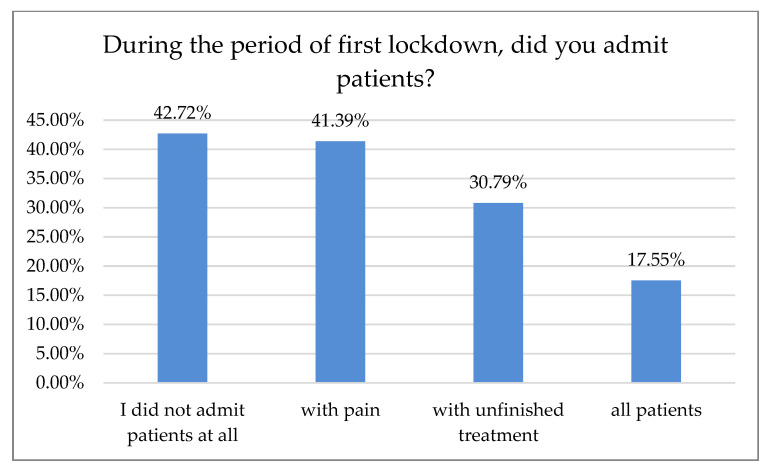
Changes in patients admitted during the lockdown.

Of the respondents, 42.72% did not admit patients during the lockdown, 41.39% admitted only pain treatments, and 30.79% admitted patients with unfinished treatment. Fifty-three dentists (17.55%) admitted all patients (Figure 8).

**Figure 9:**
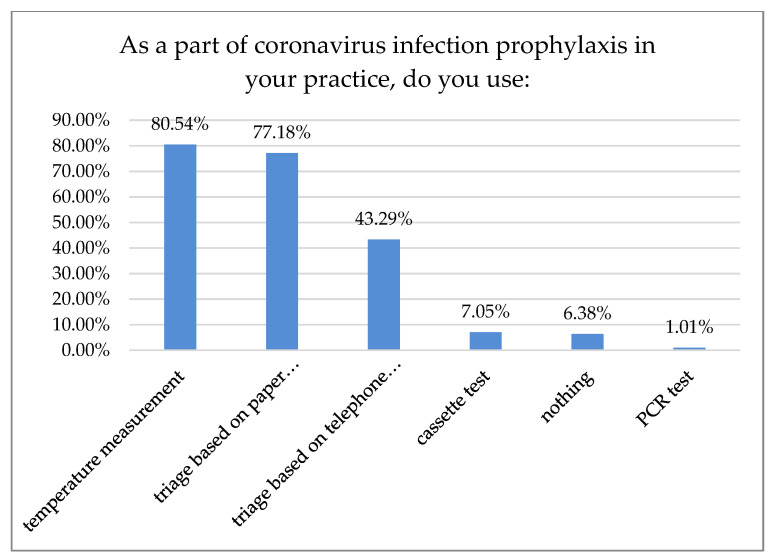
Coronavirus precautions in place of practice.

Most dentists (80.54%) reported temperature measurement as a method of prophylaxis. Of those dentists, 43.29% used triage-based telephone surveys and 77.18% demanded a signed paper survey. Only 1.01% of the surveyed dentists required a negative RT-PCR (Reverse-Transcription Polymerase Chain Reaction) test before treatment (Figure 9).

No sex effect on sleep quality before and after the pandemic was observed. However, there was a significant time effect and sex× time interaction. The decrease in sleep quality among women was more significant compared to men (Table 2).

Significant time effect without the influence of sex was found when analyzing sleep length. Both groups declared a decrease in sleep hours (Table 3).

Sex and time effect and their interaction were significant in the number of headache episodes. They were more frequent among women before the pandemic. After the pandemic, their frequency increased among both groups. However, this increase was more significant among female dentists (Table 4).

An increase in neck and back pain was observed. Women were characterized by a more significant increase in pain sensations (Table 5).

The decrease in working hours was more significant among women than men (Table 6).

Men were characterized by greater alcohol intake before the pandemic. The increase in alcohol usage during the pandemic was more significant among men than women (Table 7).

A decrease in the number of patients was associated with a price increase (Table 8).

A weak positive correlation between time spent on admitting a single patient and the number of protective devices was observed (Table 9).

The occurrence of temporomandibular disorders (TMDs) was correlated with headache frequency, amount of working hours, alcohol intake, and sleep quality (Table 10).

TMDs were more frequent among women (Table 11).

## 4. Discussion

This survey-based study aimed to evaluate the impact of SARS-CoV-2 on the Polish dental professionals population, their work, personal health, quality of life, and attitude toward providing dental care facing theSARS-CoV-2 threat. It provides initial insight into contributing factors and primary reasons that drove Polish dentists’ behavior and attitude toward the pandemic. Possible transmission-related issues and availability of PPE during the first six months of the SARS-CoV-2 pandemic were also scrutinized, including self-esteem, reported welfare, and claimed health concerns. According to the collected data, women were overrepresented—the studied group consisted of 207 women (68.32%) and 96 men (31.68%), which is consistent with information obtained from the State Registry of Healthcare Providers, as the number of female dental practitioners in Poland (77%) is higher than male dentists (23%) [15]. The mean age of the survey responders was 39.31 ± 10.43 years, and the mean length of running a dental practice was 14.31 ± 10.29 years. The place of residence distribution is described in Table 1. Just 24 of the respondents declared the countryside as their only place of practice (7.92%), whereas 279 other dentists declared working solely in cities with a population of over 100,000 inhabitants. This example is representative, as there is almost equal access to social media in Poland. Densely inhabited areas have about 92% broadband Internet connection coverage. In rural areas, roughly 89.5%of homes confirmed having a suitable quality of Internet access [16]. On the contrary, another Polish study by Tysiąc-Miśta and Dziedzic revoked a selection bias of some sort, citing social network dissemination and inequality to Internet access in the questionnaire [17].

According to 50% of our respondents, the number of treated patients decreased significantly over the last six months, while 70.7% of the respondents reported a treatment price increase within the same time period. Only 15.5% of Polish dentists declared that they had no break during the first wave of the pandemic. Most declared one- or two-month breaks during that time, 30.7% and 34.7%, respectively. In Northern Italy, dental offices were closed or highly reduced their activity to urgent procedures. However, 96.1% of the dentists guaranteed telephone availability upon request: if an emergency occurred, 45% of the respondents took care of it alone and 55% were helped by an assistant. Seventy percent of Italian dentists reported an average number of 6–15 patients a day before the outbreak, which shifted swiftly to0–5 a week in 90% of the surveyed practice owners shortly after [18]. In Switzerland and Liechtenstein, almost 50% of dentists continued their practice with increased precautions, sanitary regimes, and state-recommended measures. Significantly fewer (21%) stopped their business activities for more than two weeks, while 19% of the surveyed practitioners continued their work. However, they limited appointments to pain interventions and emergencies that could not have been postponed (19%). Almost 2% continued their work without altering daily practice, while more than 3% had to close their businesses permanently, mostly due to economic reasons. The majority of dentists had to reduce their practice activity to a minimum of 0–10% efficiency, as almost 70% surveyed reported. Of Swiss dentists, 14.6% limited their practice activity to 11–30%, and 3.8% to 31–60%, albeit 11.6% had slight to no reduction [19]. Most Polish dentists (80.54%) reported on-site temperature measurement as precautionary model prophylaxis of virus transmission. The other 43.29% were using a triage-based telephone survey. Hence, 77.18% of the dental practitioners also required a paper survey and signed written consent before each dental appointment. Only 1.01% of the examined dentists in Poland demanded a negative PT-PCR test before treatment. In Iran, where dental services are actively provided by 20,000 practitioners [20], a questionnaire-based report with 240 participants carried out in June 2020 after the first wave showed that most dentists (*n* = 210, 87%) had problems providing PPS, and 70% of them did not perform non-emergency procedures during the pandemic. Additionally, 46% of the participants canceled all dental procedures temporarily since the SARS-CoV-2pandemic outbreak, and 95% of them changed their work hours. Those who remained open (66%) asserted that the test detecting SARS-CoV-2 must be completed for all patients regardless of the type of dental procedure [21]. Dentistry uses a plethora of PPE, e.g., surgical masks, glasses, goggles, protective helmets, etc. While some of them, especially magnifying eye protection equipment, can positively influence dental treatment, they may be prone to fogging. Yet, full face coverage may impede magnification equipment, e.g., loupes or microscope usage. Some clinicians even tried to adapt or customize swimming masks and other face protection devices when the availability of professional, disposable PPE was scarce [22]. Our data revealed that 24.09% of the surveyed dentists had to cease working with magnification due to increased personal protection. While full eye coverage and disposable protection equipment had been, in most cases, the preferred countermeasure prior to virus transmission, it may affect an entire treatment process: difficulties in loupe or microscope usage were previously reported [23]. Nowadays, these tools are considered essential in dentistry for treatment accuracy and predictability improvement; according to the majority of data, precision was significantly higher when a microscope was used, followed closely by loupes [24,25]. Many other researchers reported the fear of potential exposure to the virus and concern about availability vs. high demand for dental PPE, including the increasing prices of disposable medical equipment [26,27,28,29,30,31,32].

In our survey, headache episodes were more frequent among female dentists before the pandemic. After the pandemic began, headache frequency increased among both sexes; however, this increase was even more significant among female dental practitioners (Table 4). TMDs were also more frequent among women, according to data we obtained (Table 11). Prevalence of TMD-related pain and severity was reported twice as often in women than in men [33]. Some studies evaluated the potential role of sex on TMD pathogenesis by investigating sex hormones such as estrogen. This hormone is shown to play an essential role in the symptomatology of female-predominant TMDs, synovitis, chondrocyte pathology, and orofacial pain [34]. Myofascial pain is about three times more common in women than in men and is mostly reported among TMD patients (45.3%), patients suffering from temporomandipular joint TMJ disk displacement (41.1%), or patients with TMJ arthralgia (34.2%) [35]. These data seem to correlate with this survey, as the study group consisted of the majority of women (68.32%) and 96 men (31.68%). Correspondingly, an increase in neck and back discomfort was observed. Female dentists were characterized by a more significant increase in pain, as shown in Table 5. The study from Saudi Arabia conducted between May and August 2020, based on the Pittsburgh Sleep Quality Index (PSQI) scale among doctors in the age range of 31–40 years, showed an increase in the prevalence of sleep disorders (63%) compared to other age groups. Additionally, dentists were among different medical specialties in the group of professionals with the highest prevalence (66.7%) of sleep disorders, just after anesthetists and laboratory/pathology/microbiology specialists [36]. In a similar Dutch study by Kocevska et al. [37], profound individual differences in the effect of the lockdown measures on the perceived sleep quality were found. While some study subjects experienced improved sleep quality, a portion of the examined patients experienced a decrease in sleep quality during the pandemic. They also observed that the severity of negative thoughts and worrying experienced through lock down was related to the change in self-reported sleep quality. Improved processing of such data, possible in larger cohorts, may lead to a better understanding of sleep problems and deteriorating mental health in these trying times.

One in six adults in the United Kingdom increased their alcohol intake during the lockdown, especially younger adults. This is partly consistent with our findings. In this study, male respondents were characterized by greater alcohol intake before the pandemic. The increase in alcohol usage during the pandemic was more significant among men than women [38], which is shown in Table 7.

One of the limitations of this study was the relatively short period of data acquisition. Yet, similar papers published covered a similar timeframe [17,18,19,20,21]. This study was conducted only among dentists who had access to social media, which likely influenced the overall number of respondents.

## 5. Conclusions

Based on the conducted research, most dentists in Poland have followed SARS-CoV-2 recommendation protocols and have restricted their practices to admitting only patients with pain or incomplete treatment. That, together with a one- or two-month freeze of their businesses, may be a reason why a relatively small proportion of dentists (*n* = 41) reported infection with SARS-CoV-2. The most popular infection precautions were temperature measurement and telephone or paper questionnaires, resulting from limited access to a quick antigen or PT-PCR tests.

Sleep parameters such as quality and number of hours have decreased during the pandemic. Head, back, and neck pain were common symptoms. All these issues, and increased alcohol intake, are clearly correlated to TMD. As the number of patients decreased and prices increased, the economic influence of the pandemic on this branch of the health care system should be carefully monitored by public organizations to support dentists both financially and psychologically.

## Figures and Tables

**Table 1:** The profile of performed procedures.

The Main Profile of Performed Procedures		
	*n*	%
Other	11	3.64%
Conservative dentistry	244	80.79%
Endodontics	153	50.66%
Dental surgery	106	35.10%
Prosthodontics	149	49.34%
Orthodontics	30	9.93%
Periodontology	44	14.57%

**Table 2:** Sleep quality before and during the pandemic.

	Women		Men	
	Mean	SD	Mean	SD
What was your sleep quality before March 2020 on a scale from 0 to 10?	7.66	1.61	7.46	1.42
What was your sleep quality after the introduction of restrictions and the outbreak of the pandemic after March 2020 on a scale of 0–10?	6.00	2.16	6.77	1.73
Women vs. Men	0.142	time <0.0001	time× sex	<0.0001

**Table 3:** Sleep timespan before and during the pandemic.

	Women		Men	
	Mean	SD	Mean	SD
Number of hours of sleep per day before March 2020 from 1 to 10	7.19	0.92	6.92	1.01
Number of hours of sleep per day after March 2020 from 1 to 10	6.85	1.23	6.72	1.11
Women vs. Men	0.066	time <0.0001	time× sex	0.324

**Table 4:** Headache episodes among dentists before and during the pandemic.

	Women		Men	
	Mean	SD	Mean	SD
Episodes of headaches before March 2020 on a scale from 0 to 10	3.01	2.16	2.18	2.19
Episodes of headaches after March 2020 on a scale from 0 to 10	4.23	2.68	2.90	2.59
Women vs. men	0.0001	time <0.0001	time× sex	0.049

**Table 5:** Neck pain episodes before and during the pandemic.

	Women		Men	
	Mean	SD	Mean	SD
Neck/back/spine pain before March 2020 on a scale from 0 to 10	4.20	2.42	3.46	2.78
Neck/back/spine pain after March 2020 on a scale from 0 to 10	5.22	2.67	3.65	3.00
Women vs. Men	0.0002	time <0.0001	time× sex	0.001

**Table 6:** Working hours before and during the pandemic.

	Women		Men	
	Mean	SD	Mean	SD
The average number of working hours per day before March 2020 on a scale from 1 to 10	7.36	1.51	7.99	1.78
The average number of working hours per day after March 2020 on a scale from 1 to 10	6.69	1.83	7.08	2.00
Women vs. Men	0.005	time <0.0001	time× sex	<0.0001

**Table 7:** Alcohol intake among groups.

	Women		Men	
	Mean	SD	Mean	SD
Alcohol consumption or other stimulants intake before March 2020 on a scale from 0 to 10	2.01	1.53	3.49	1.91
Alcohol consumption or other stimulants intake from March 2020 on a scale from 0 to 10	2.48	2.08	4.39	2.23
Women vs. men	<0.0001	time <0.0001	time× sex	0.011

**Table 8:** Association between the number of patients and price change during the pandemic.

Has the Number of Patients over the Last Six Months?	*n*	%	*n*	%	*n*	%	*p*
	Prices Increased		Prices Not Changed		Prices Decreased		
Decreased	127	59.07%	37	43.53%	3	75.00%	0.033
Not changed	65	30.23%	39	45.88%	0	0.00%	
Increased	23	10.70%	9	10.59%	1	25.00%	

**Table 9:** Correlation between time spent on admitting a single patient and the number of protective devices.

Pair of Variables	Tau	*p*
Number of patients and number of protective devices	0.04	0.29
Changes in prices during the last six months and number of protective devices	0.06	0.11
Amount of time spent admitting single patient and number of protective devices	0.10	0.01

**Table 10:** Correlation between TMD intensity and other associated factors.

Pair of Variables	Tau	*p*
TMD and sleep quality	−0.25	0.00
TMD and headaches frequency before the pandemic	0.13	0.00
TMD and headaches frequency after the pandemic	0.20	0.00
TMD and back and neck pain during the pandemic	0.13	0.00
TMD and mean number of working hours before the pandemic	0.09	0.02
TMD and alcohol intake during the pandemic	0.08	0.03
TMD and body weight during the pandemic	0.09	0.03

**Table 11:** Association between TMDs and sex.

Have You Experienced any TMD problems in the Period Mentioned Above?	*n*	%	*n*	%	*p*
	Women		Men		
I do not know	3	1.45%	1	1.05%	0.017
I do not think so	34	16.43%	16	16.84%	
No	84	40.58%	52	54.74%	
I think so	45	21.74%	21	22.11%	
Yes	41	19.81%	5	5.26%	
Total	207		95		

## Data Availability

The data presented in this study are available on request from the corresponding author. The data are not publicly available due to sensitive information.

## Supplementary Materials

The following are available online at https://www.mdpi.com/1660-4601/18/3/1281/s1, Supplement 1 and Supplement 2.
