# Supplementary material for: The Impact of SARS-CoV-2 Outbreak on the Polish Dental Community’s Standards of Care—A Six-Month Retrospective Survey-Based Study
Source: Int J Environ Res Public Health. 2021 Jan 31;18(3):1281. doi: 10.3390/ijerph18031281 (PMC7908176; doi:10.3390/ijerph18031281)
Supplement: Supplementary file 1 [file ijerph-18-01281-s001.pdf]

Supplement 1.

Age structure of Polish dentists.

age	male	female	total
24-35	2797	7369	10166
36-45	2195	5739	7934
46-55	1195	6662	7875
56-65	1409	5319	6728
65+	1297	7951	9248
total number of licensed dentists		9923	33040
number of active dentists		9515	28972

Supplement 2.

*questions where more than one answer was possible

1. sex

male female

2. age

3. number of years you've worked

4. place of residence

City up to 100 000 citizens

City above 100 000 citizens

countryside/ village

5. place of practice

City up to 100 000 citizens

City above 100 000 citizens

countryside/ village

*6. main profile of performed procedures

conservative dentistry

endodontics

periodontology

orthodontics

dental surgery

craniofacial surgery

esthetic medicine

prosthodontics

dental implantology

pediatric dentistry

other

7. has the number of patients over last 6 months

decreased not changed increased

8. have the prices of provided services over last 6 months

decreased not changed increased

9. Did you have a break during the first wave of pandemic

no

up to 1 month

up to 2 months

up to 3 months

up to 4 monthss

up to 5 months

I haven`t seen patients at all for the last 6 months

*10. During the period of first lockdown did you admit

patients with pain

patients with unfinished treatment

I did not admit patients at all

all patients

11. Due to personal protective equipment used, did you have to give up working with magnification (magnifiers, microscope) or limit their use:

Yes

No

I do not use magnification

12. The amount of time you spend admitting a single patient compared to before pandemic

decreased has not changed increased

13. Have you been (or are) infected with SARS-COV2 ?:

Yes No

*14. Have you worked during this period with:

no mask

surgical mask

FFP2/N95 mask

FFP3 mask

full face mask

HAZMAT

glasses

googles

protective helmet

*15. As a part of SARS-COV2 infection prophylaxis in your practice, you use:

nothing

triage based on telephone survey

triage based on paper questionnaire

temperature measurement

cassette test

PCR test

16. Have you experienced any TMJ disorders in the above-mentioned period such as clenching/grinding, muscle pain in stomatognathic system, generalized hypersensitivity of enamel or dentin

that did not occur before, or any symptoms that occurred before?

Yes

No

I think so

I don't think so

I don't know

17. If you suffer from any chronic disease have you observed any of the following

exacerbation/ aggravation of symptoms

nothing has changed

decrease or resolution of symptoms

I don't suffer from any chronic disease

18. what was your sleep quality before March 2020 on a scale from 0 to 10 where 0- insomnia and 10- perfect sleep?

19. What was your sleep quality after introduction of restrictions and the outbreak of the pandemic after March 2020 on a scale 0-10

20. Number of hours of sleep per day before March 2020 from 1 to 10

21. Number of hours of sleep per day from March 2020 from 1 to 10

22. Episodes of headaches before March 2020 on a scale from 0 to 10 where 0- never 10- everyday

23. Episodes of headaches from March 2020 on a scale from 0 to 10 where 0- never 10- everyday

24. Neck/back/spine pain before March 2020 on a scale from 0 to 10 where 0- never 10- everyday

25. Neck/back/spine pain from March 2020 on a scale from 0 to 10 where 0- never 10- everyday

26. An average number of working hours per day before March 2020 on a scale from 1 to 10

27. An average number of working hours per day from March 2020 on a scale from 1 to 10

28. Alcohol consumption or other stimulants intake before March 2020 on a scale from 0 to 10 where 0- never, 10- everyday

29. Alcohol consumption or other stimulants intake from March 2020 on a scale from 0 to 10 where 0- never, 10- everyday

30. Has your body weight changed compared to before pandemic

Has not changed

Increased

Decreased

I don't know- I don't control my bodyweight
